# Making community pharmacies psychologically informed environments (PIE): a feasibility study to improve engagement with people using drug services in Scotland

**DOI:** 10.1017/S1463423623000087

**Published:** 2023-03-16

**Authors:** Catriona Matheson, Carole Hunter, Joe Schofield, Kate O’Sullivan, Janie Hunter, Alison Munro, Tessa Parkes

**Affiliations:** 1 Drugs Research Network for Scotland, University of Stirling, Stirling, UK; 2 NHS Greater Glasgow and Clyde Health Board, Glasgow, UK; 3 Faculty of Social Sciences, University of Stirling, Stirling, UK; 4 School of Health Sciences, University of Dundee, Dundee, UK

**Keywords:** drug use, pharmaceutical care, pharmacy, psychologically informed environments, trauma

## Abstract

**Aim:**

This developmental study tested the feasibility of training pharmacy staff on the psychologically informed environments (PIE) approach to improve the delivery of care.

**Background:**

Community pharmacies provide key services to people who use drugs (PWUD) through needle exchange services, medication-assisted treatment and naloxone distribution. PWUD often have trauma backgrounds, and an approach that has been demonstrated to work well in the homeless sector is PIEs.

**Methods:**

Bespoke training was provided by clinical psychologists and assessed by questionnaire. Staff interviews explored changes made following PIE training to adapt the delivery of care. Changes in attitude of staff following training were assessed by questionnaire. Peer researchers interviewed patient/client on observed changes and experiences in participating pharmacies. Staff interviews were conducted six months after training to determine what changes, if any, staff had implemented. Normalisation process theory (NPT) provided a framework for assessing change.

**Findings:**

Three pharmacies (16 staff) participated. Training evaluation was positive; all participants rated training structure and delivery as ‘very good’ or ‘excellent’. There was no statistically significant change in attitudes. COVID-19 lockdowns restricted follow-up data collection. Staff interviews revealed training had encouraged staff to reflect on their practice and communication and consider potentially discriminatory practice. PIE informed communication skills were applied to manage COVID-19 changes. Staff across pharmacies noted mental health challenges for patients. Five patients were interviewed but COVID-19 delays in data collection meant changes in delivery of care were difficult to recall. However, they did reflect on interactions with pharmacy staff generally. Across staff and patient interviews, there was possible conflation of practice changes due to COVID-19 and the training. However, the study found that training pharmacy teams in PIE was feasible, well received, and further development is recommended. There was evidence of the four NPT domains to support change (coherence, cognitive participation, collective action and reflexive monitoring).

## Background

Community pharmacies are a key healthcare service for people who use drugs (PWUD), providing specialist harm reduction, and treatment interventions such as naloxone, injecting equipment provision, medication-assisted treatment (MAT: care including the provision of methadone or buprenorphine) and blood borne virus testing and treatment (Matheson *et al.* 2016; Scott *et al.* 2017; Sheridan *et al.* 2007; Matheson *et al.* 2002).

There is strong evidence documenting significantly improved attitudes among the population of community pharmacy staff across Scotland towards PWUD and services for PWUD from 1995-2015 (Matheson *et al.* 2016). However, there are anecdotal reports of people experiencing barriers to access and perceptions of being treated differently to other pharmacy patients. This suggests a difference in expectations between service providers and PWUD. As training generally has a significant positive impact on pharmacy staff attitudes, new training focussing on the vulnerabilities of PWUD was considered. An approach gaining success in other sectors for example homeless services is that of a psychologically informed environment (PIE) (Johnson & Haigh, 2010; Maguire *et al.* 2010).

PWUD are disproportionately burdened with mental ill-health and psychiatric conditions and frequently report a history of complex trauma (Couto *et al.* 2018; Reddon *et al.* 2018; Wang *et al.* 2016). Complex trauma is characterised by repeated interpersonal traumas, such as childhood abuse or domestic violence, and is a strong risk factor for problematic drug use (Johnson & Haigh, 2010; Konkolÿ-Thege *et al.* 2017). These conditions can be exacerbated by experiences of stigma and discrimination from health and care providers, resulting in mistrust, fear of engaging with services and suboptimal engagement rates (Edlin *et al.* 2005; French *et al.* 2011; Hyshka *et al.* 2017; PHE, 2019). Engagement in harm reduction and treatment services is protective against drug-related morbidity and mortality (Degenhardt *et al.* 2011). There is evidence that suboptimal treatment uptake and an ageing cohort of PWUD with multiple comorbidities are contributing to increasing in drug-related deaths in Scotland (1264 in 2019, from 497 in 2007), (NRS, 2020). Older PWUD have reported isolation and loneliness and high levels of depression and anxiety (Matheson *et al.* 2018).

A PIE approach is characterised by ‘trying to understand people’s behaviour, helping them to be involved with others in a genuine way, and to take as much responsibility for themselves as possible’ (Maguire *et al.* 2010). The core elements of a PIE are creating a space which engenders a sense of safety and wellbeing; reflective practice so staff can develop a shared model of working across staff; training and support for staff; and considering the therapeutic aspects of service provision to vulnerable people (Johnson & Haigh, 2010). The PIE movement aims to create enabling environments and can be flexibly applied in a range of settings. PIEs have been explored in settings that work with vulnerable populations with longstanding mental health problems, psychological needs and emotional problems, primarily homeless and criminal justice services (Breedvelt, 2016). Fundamental to a PIE is understanding the impact of trauma on affected people. Experience of trauma is common among PWUD (Huggard *et al.* 2017). Whilst there is a growing body of research on PIEs (Breedvelt, 2016), a search of published and grey literature found no reports on this approach within community pharmacy or its applicability to PWUD. The concept of psychologically informed pharmacies could have relevance well beyond the UK and beyond the target group of PWUD.

This study aimed to test the feasibility of training whole pharmacy staff teams in PIE and to explore changes in the delivery of care as a result of this training intervention. The hypothesis was that training staff in PIE might encourage a review of, and change in, delivery of care that could improve engagement, retention and ultimately treatment outcomes for PWUD. This developmental study focussed on the feasibility of training and changes to practice only.

### Methods

This mixed method study collected data via questionnaires and interviews. Normalisation process theory (NPT) provided a framework for evaluating the PIE training intervention. This was considered appropriate because of the focus on change implementation (May & Finch, 2009). NPT has four constructs: coherence, cognitive participation, collective action and reflexive monitoring (May & Finch, 2009). Coherence refers to the process of understanding that individuals and organisations (eg, a pharmacy) go through to understand the new approach/intervention. Cognitive participation involves enrolling and engaging individuals into new practices. Collective action is the work done to embed the intervention into routine practice. Reflexive monitoring refers to formal and informal intervention appraisal. NPT facilitated data collection and analysis of how pharmacy staff perceived a PIE approach, how PWUD experience the PIE pharmacy and other factors impacting on delivery and routinisation.

The project was considered evaluation and as such did not require NHS ethical approval (see ethical statement).

### Recruitment of pharmacies and pharmacy staff

The study was conducted in one council area within NHS Greater Glasgow and Clyde (GGC) Health Board for logistical reasons given the large size of GGC. Author (CH), Lead Pharmacist for Addiction services, recruited three high-volume (> 20 MAT patients) pharmacies who also provide injecting equipment. CH reviewed eligible pharmacies, and a purposive sample was selected to cover a range of type of pharmacy, size and location. CH contacted pharmacies initially to see if they were interested in participation. The lead researcher then provided written and verbal study information to each pharmacist manager and their professional support services. All staff were invited to participate in one of two training sessions. Participation was voluntary. Participation involved completion of a questionnaire immediately before training and at six months after attending a training session and a short interview at six months post-training.

### Training pharmacy staff in PIE (the intervention)

The training programme was designed by KO’S, JH, CH and CM such that it would be suitable for a range of pharmacy personnel, with different levels of education and experience. Training was delivered through a two-hour evening session delivered by clinical psychologists (KO’S and JH) experienced in trauma-informed care and supported by printed information on the study, PIE and key local services. Two evening sessions were held in February 2020.

Part 1 of the training session covered ‘Understanding the impact of trauma’, which included (i) the context of psychologically informed working, (ii) PIE, (iii) What is trauma? And (iv) how do we see the impact of trauma in our patients?

Part 2 of training covered ‘Putting PIE into practice’ and included consideration of interpersonal communication, environment and processes. Participants spent time discussing their current practice and areas in which they might be considered a change in practice.

### Evaluation of impact on staff

A validated questionnaire was used to measure staff attitudes to people with a drug problem and pharmacy-based service provision for PWUD. This validated questionnaire has been used in four national surveys of all community pharmacies in Scotland undertaken by the lead researcher (Matheson *et al.* 2016). The attitude scale component of the questionnaire was used which comprised 26 statements and a 5-point Likert scale which is totalled to produce a score (range –52 to 52) with any score over zero representing a positive attitude. Baseline questionnaire completion was undertaken immediately prior to training. The same questionnaire was completed six months after training. Questionnaires were pseudonomised.

Staff feedback was sought by face-to-face or telephone interview by the lead author (CM), a researcher with over twenty-five years of conducting qualitative interviews and mixed methods research. The topic guide used the broad constructs of coherence, cognitive participation, collective action and reflective monitoring to explore: changes in practice informed by a PIE approach; participation of staff in driving changes to practice; involvement of patients/clients in any changes; maintaining changes and moving forward; application to other patient groups.

### Patient/Client data collection

Patients were recruited via staff as it was not possible to have researchers in the pharmacy due to COVID-19 restrictions. Semi-structured interviews, conducted by trained peer researchers, explored whether patients had perceived changes in the way the service was delivered. This was conducted in an exploratory way and deliberately did not explain to participants that staff had had extra training. Due to COVID-19 restrictions, researchers could not attend pharmacies so pharmacy staff helped recruit patients for telephone interviews with researchers. The peer researchers who conducted interviews volunteered for Scottish Drugs Forum (SDF) as part of their volunteer training programme. Researchers received training in data collection and were supervised and supported by a member of SDF staff to organise and conduct data collection. Pharmacy staff liaised directly with the SDF coordinator to arrange telephone calls between patient and peer researcher.

### Data analysis

Quantitative analysis explored change in attitude score between baseline (pre-training) and six month follow-up using dependent-samples sign-test, with a 5% significance level. Qualitative data from interviews were analysed using both deductive and inductive approaches. A deductive analysis was undertaken in which the dataset was considered within the broad NPT concepts of coherence, cognitive participation, collective action and reflexive monitoring (May & Finch, 2009). In addition, emerging themes were incorporated into a thematic framework and applied across the dataset. The range of views and experience under themes are described using illustrative verbatim quotes.

## Results

### Pharmacy and staff participation

Three pharmacies participated with a total of seventeen staff members. All but one were female. Pharmacies included two large national and one small local chains from a town centre, high street and housing estate. Training participation reflects the NPT concept of coherence. Sixteen of a possible 17 staff attended training: three pharmacists, one pre-registration pharmacist, four dispensers, six pharmacy technicians and two counter assistants. All three pharmacists left their respective pharmacies between the training and follow-up. Follow-up data (attitude questionnaire and interview) were collected from two of these pharmacists before leaving.

### Training evaluation: psychologically informed pharmacies

Training evaluation reflects the NPT concept of cognitive participation. Training was very well received with 15/16 expressing the highest score in general satisfaction. Speed of delivery and level of difficulty were considered optimal by 15 participants (1 missing). Detailed evaluation data are presented in Tables 1–3. Only three participants made any comments in the free text box. These are listed verbatim: i) ‘*was every good training session, thoroughly enjoyed the chat & insight into why training effects People. Staff very informative :)’* ii) ‘*Very valuable information, more & more patients coming through the pharmacy with trauma so interesting to know more about it’*, iii) ‘*Great session’*.

**Table 1.** Training content (*n* = 16)

	Strongly disagree	Moderately disagree	Slightly disagree	Slightly agree	Moderately agree	Strongly agree
General acceptability: This approach would be appropriate for a variety of staff				1	3	12
Effectiveness: The training would be beneficial for the staff					3	13
Negative side effects: The training will result in disruption or harm to clients	13	2				1
Appropriateness: Most staff would not accept that the training provided an appropriate approach to client care	13	1		1	1	
Consistency: The training was consistent with common sense and good practice in helping staff to work effectively					2	14
Social Validity: In an overall, general sense, most staff would approve of training in this method					3	13

**Table 2.** Teaching process and outcomes (n = 16)

	Not at all	A little	Quite a lot	A great deal
Did the workshop improve your understanding?		2	1	12
Did the workshop help you to develop work-related skills?		2	3	11
Has the workshop made you more confident?		3	3	10
Do you expect to make use of what you learnt during the workshop in your workplace?		1	2	13
How competent were the workshop leaders?				16
In an overall, general sense, how satisfied are you with the workshop?			1	15

**Table 3.** Training structure and delivery (*n* = 16)

	V poor 1	2	3	4	5	Excellent 6
clear aims & objectives	–	–	–	–	3	13
well-planned & organised	–	–	–	–	2	14
effective use of time	–	–	–	–	3	13
clarity of content	–	–	–	1	2	13
use of visual aids	–	–	–	–	2	13
Handouts^*^	–	–	–	–	5	5
level of group participation	–	–	–	–	3	13
good use of examples	–	–	–	–	2	14

* Six missing.

### Attitude measurement

The measurement of changes in attitudes to this patient/client group reflected the NPT concept of ‘reflective monitoring’. All seventeen participants completed the baseline attitudinal questionnaire, of whom 11 completed a follow-up questionnaire. At baseline, all attitudinal scores were positive (> 0) with a median of 15.0 (IQR 11.25). Post-intervention, the median score was 20 (IQR 11.50). There was no evidence of a significant difference in scores (*S* = 4, *P* = 0.549).

### Pharmacy staff follow-up interviews

Staff interviews included two pharmacists (by telephone three months post-training) and in-person interviews with ten additional staff (pharmacy technicians, dispensers and counter staff) at six months representing 12/16 of trained staff.

### Changes to practice following training

The changes made to practice following training reflected the NPT concept of ‘collective action’, so staff were specifically asked what changes they had made following the training. Different pharmacies focussed on different areas of practice. In one pharmacy, staff consulted patients straight after the training. Staff noticed differences in the use of first names across patient groups at the training (noting the common use of first names with PUWD attending the pharmacy, but not with other patient groups). Whilst they reported doing this in order to be ‘friendly’, following discussion they realised they should ask all clients how they prefer to be addressed. This more person-centred practice was new to many pharmacy staff:

*‘The only thing that’s kind of stuck in my mind from when we had the meeting [training] was about how, we shout names, so we’ll shout first name, and we spoke about that in [the] meeting, that to maybe ask them, because sometimes they maybe don’t want you to shout their first names out, and then there’s patient confidentiality as well, so that just something I wasn’t really aware of before, just because you think you’re being friendly to them’.*

In many pharmacies, staff reported asking PWUD receiving MAT to avoid attending at specific times, for example, at opening time (to allow staff to prepare for MAT dispensing) or at busier times of day. Staff at one participating pharmacy felt this remained beneficial as it allowed them to reduce patient waiting times, especially during COVID-19. Another pharmacy reported changing this practice, noting they did not restrict dispensing times for other patient groups. Awareness of discrimination was evident in other comments:

*‘ I do think like we get patients in coming and saying, they get treated like rubbish … and they [staff in other pharmacies] just don’t care, so actually thinking about them, like what they’ve gone through, and what they’re thinking like, it takes 5 min to listen…it’s not that long, a wee old woman would come in and you listen to them, so why should it be different’.*

Training had increased awareness of patient experiences of discrimination and trauma-informed changes to communication with patients.

### Physical changes to the pharmacy environment

Regarding physical changes to the pharmacy environment following training, in one pharmacy, staff started to ask patients if they wanted to use the private area or not for consumption of their medication. Before training everyone was taken into the private area, which, after training, staff realised could single patients out and potentially be a form of discrimination. There was a perception this was valued by patients who were given the choice. One participant noted she had been thinking about information sources and posters to ensure these were appropriate:

*‘having obviously, all your posters on ice [due to* COVID*], like obviously with your safer injecting, and all that, like making sure obviously the right information was there for them to read like, and we always have had up in the wee bit [private area] like all the proper kind of forms and posters, if they need obviously any help’.*

The emerging themes when discussing changes to practice were communication with patients/clients and COVID-19 support with sub-theme of mental health support. These are presented below.

### Communication

Although all staff interviewees raised the topic of communication, they were generally keen to highlight that they already felt they had a very positive relationship with their patients before the training.

*‘we’ve got a really good relationship, we’re really lucky with our patients’.*

However, there was a sense that the training had enabled them to reflect further on their communications with patients and customers. Staff participants did not always think they needed to make substantial changes but there was evidence of an enhanced awareness of need in patients.

*‘……I think we’ve been a wee bit more conscious lately like giving them that wee extra bit of time, just because they know how hard things are for everybody, like with the* COVID*, the lockdown, maybe spend that wee bit extra time on the phone to the elderly, and they are thankful for it, like, they do tell you that’.*

Another pharmacy had made simple changes around communication with people if they have to wait for medication to explain why people might have to wait. The awareness of the behaviour of clients, in this case agitation, was attributed to the training:

*‘What we took from the training was to tell the methadone patients what we were doing at the time, because that, that was the main priority in the pharmacy, it was because they were getting more agitated, because we’re [no] telling them what we’re actually doing and they were left standing there when we were busy’.*

Some staff reflected on the deeper relationship with some patients that developed over time allowing them to anticipate and read body language:

*‘… you can tell by their body language if they, they’re going to be up for talking today or not, so you kind of have that as well, because with some of them, because I’ve been here a while, I kind of know some of them have gotten worse, so some of them are getting better, so then you can have that conversation………’*

There was also an awareness of the importance of responding to patients’ additional support needs, for example, reminders about appointments and prescription renewal.

***‘**To be honest, we’ve always said to people, like if you need us, if you need a talk, you need any extra help, they could come to us’.*

Increased trauma awareness had made staff more aware, and they discussed amongst themselves the possibility of trauma for particular patients:

*‘Yeah, I think a lot of the staff have kind of been more aware of just, because you still kind of speak about it here,.…what trauma they could have gone through to, you know, [to] be the way they are, kind of, and it helps them, or even now, I mean a lot of the patients will have that kind of conversation with us about their day-to-day kind of difficulties as well, or their personal stories, and a lot of the time, it’s difficult to take, to listen to that and then have that kind of on you, but the rest of us are here to kind of listen to each other’.*

This raised the issue for staff of what to do with some of the disclosed information. However in this case, they used each other as a source of support in processing difficult stories from their patients reflecting the importance of shared training and shared values.

### COVID-19 support

The impact of COVID-19 was a common thread throughout interviews and affected the project as pandemic restrictions came in after the training when pharmacy staff were discussing PIE changes:

*‘ …like it’s just dead hard like, because we never got a chance to do a big, like big changes obviously, within the 2 weeks of the meeting like it was just all* COVID*’.*

However, the timing of the training and follow-up allowed the opportunity to ask staff participants whether the training had helped manage issues around COVID-19. The strong emerging theme was that of the additional support they provided for patients experiencing mental health problems due to COVID-19 restrictions. This was not just for their patients attending for drug-related problems and extended to older people. Staff were clearly acutely aware they might be the only person someone sees or speaks to that day. Participants noted their patients were not having the usual face-to-face contact with their drugs workers and some could not access their GP.

*‘So we have noticed, particularly because of the* COVID *stuff, mental health has gone through the roof, so more and more, this past sort of maybe 2, 3 months, there’s been a massive increase in people’s mental health problems…. They’re actually coming and talking to us about it, yeah, and because there’s no access directly to GP’s, we’re the sort of first people that people are seeing……and quite a few of our daily patients, we’ve saw a massive decline in their mental health as well, which is quite alarming’.*

When asked how they handled that:

*‘We’ve just been talking to people, we’ve been letting people know that they’re not alone, that they can pick up the phone, come in and speak to us if they’re worried about something, we’ve just been sort of pointing people in direction of websites, if we can, because a lot of things is online now, isn’t it’*

This themes of increased awareness of mental health was raised by participants in every pharmacy.

### Extending PIE to other patient groups

Under the NPT theme of collective action, participants were asked whether training had made them think differently about other patient groups and whether they applied it more widely. This again led to discussion around other patients with mental health problems, often related to COVID-19.

*‘Mental health, aye no, definitely, like the training obviously like we’re talking about the different groups that they could go for some help, and stuff like, and then I just think obviously with* COVID *as well’.*

The other patient group mentioned in these discussion were older people. This was again related to the need for contact and support, particularly during COVID-19 restrictions.

*‘…we are always here to help them any way we can, even if it was like a wee elderly patient that’s missed their delivery, we would take it out to them, so we had, all during* COVID*, when the elderly patients were struggling to try and come out, and like shielding, we would try and help, ….we would drop their prescriptions off on the way home, we would try anything to help anybody, mental health as well’.*

These groups are not mutually exclusive as older people and PWUD in general may experience similar mental health support needs.

### Experiences of patients

Five interviews were conducted with patients receiving MAT from two pharmacies. Unfortunately, the gap between the training/practice changes and interviews (7-12 months), and the considerable impact of COVID-19 restrictions on service provision, made it very difficult for patients to accurately recall their pre-pandemic experiences of community pharmacy, despite the researchers’ efforts to ask participants to distinguish between COVID-19 and any other changes. However, participants did provide insight into how they felt they were treated in participating pharmacies before and during COVID-19 and how this compared to other pharmacies they had used in the past.

*‘…but the guy’s … don’t mind going into the [pharmacy name] because they get treated decently’.*

When asked if anything had changed, one participant noted changes to her prescription due to COVID-19 – from daily to weekly methadone dispensing:

*‘…what I mean, nothing has changed, see because they don’t see you every day, nothing changed, I mean I go in once a week, and they’re ‘hi [name], and just have a wee seat’, do you know what I mean, if somebody is in, in front of me, do you know what I mean, so nothing has changed, it’s not as if everything’s turned off, they’ve no seen you, do you know what I mean, it’s just, you’re just treated the same way as [if] you were going daily, I don’t know if it was better than it was, but it was good, the lassies training, but I’ve never seen a chemist so good with methadone users’*

## Discussion

### Summary of main findings

This developmental, feasibility study found that PIE training for community pharmacy teams was feasible and acceptable. There was evidence of the four main constructs of NPT, key to implementation and embedding new practice: coherence, cognitive participation, collective action and reflexive monitoring in participating pharmacies. Training was well received. Staff attitudes to PWUD (reflexive monitoring) were initially positive and did not significantly improve, possibly due to the small sample. Attitudes were positive. Some simple practice changes were described, and the importance of communication was emphasised. COVID-19 affected delivery of some practical practice changes as well as data collection. However, the PIE training enabled staff to recognise and respond to patient needs due to COVID-19 restrictions.

### Strengths and limitations

A strength and unique feature of the study is the whole pharmacy approach taken, which is fundamental to a PIE model. As this was a feasibility study, participant numbers were deliberately low, limiting generalisability. COVID-19 measures were introduced soon after training which hugely impacted pharmacies ability to deliver routine care and make any PIE-related practice developments. Pandemic-related social restrictions also delayed data collection and precluded face-to-face patient interviews. Consequently, patient recruitment was affected as it relied on already busy staff. Pharmacy participation was targeted at those with a large number of MAT patients who also provide injecting equipment, so pharmacies with more positive attitudes towards this patient group were more likely to want to participate. However as the group with most service delivery contact, it is these pharmacies that will benefit from PIE training, even if attitudes are already positive. This is because the training encouraged reflective changes to practice and the environment.

## Discussion of main findings

Training was tailored to the pharmacy situation and delivered at a time and place that enabled attendance. Evaluation was extremely positive suggesting considerable buy-in from staff, that is, there was ‘*coherence*’ by attending training, who acknowledged the need for service changes for PWUD, and a willingness to learn. Staff requested additional advanced PIE training (demonstrating *‘reflexive monitoring’,* that is, a willingness to continue to develop skills) and suggested this should be more widely available. Future training models could focus on local teams to encompass the whole pharmacy, as this training was, or in future, built into existing NHS national pharmacy staff training or undergraduate training. Staff described good relationships with PWUD which can result in exposure to difficult and potentially upsetting disclosures about patients’ traumatic experiences. There is considerable literature on compassion fatigue and burnout in those working as counsellors in the drug and alcohol field (Huggard *et al.* 2017) but not in the pharmacy workforce. Debriefing is routine practice in many mental health services as part of supervision, particularly following an unusual incident, but not common practice in pharmacy. Other research into the impact of ‘untrained’ people listening to distressing stories found it was distressing for the recipient. This was affected by the time spent with the person and reduced if they responded with a more distant ‘advice giving’ approach rather than a ‘validating’ one (Lewis & Manusov, 2009). Research into the impact of client disclosure of suicidality showed that many pharmacy staff report feeling uncomfortable and untrained to deal with such disclosures (Murphy *et al.* 2020). Further training should equip staff to receive and manage distressing information including that relating to complex trauma, self-harm and suicide.

The greatest changes made related to communication and awareness of possible discriminatory practices, for example, time restrictions for attendance. One pharmacy explained to patients the reasons for delays in dispensing. Another offered their patients use of the private area for supervised consumption and asked what they preferred to be called. These were simple changes. ‘C*ollective action’* was evidenced as staff teams discussed what and how they could implement changes in their pharmacy. Unfortunately, due to the almost simultaneous introduction of pandemic restrictions, it was not clear from patient interviews whether these changes were recognised by patients.

Staff mentioned extending the PIE approach to patients with mental health problems and older people, especially in the context of COVID-19 which clearly impacted on many patients’ mental health across demographics and conditions. Staff identified increasingly isolated patient groups and recognised their patient-facing support role. This further demonstrated ‘*collective action’* as staff translated learning to other patient groups.

Involvement of the whole pharmacy team ‘buy-in’ is crucial to a PIE approach as change needs to be consistent and agreed by the team to be able to deliver an enabling environment. This small study in three pharmacies found all pharmacy teams embraced the opportunity to attend training together and discuss potential changes together as a collective group. Only one member of staff in one pharmacy did not attend the training. Of note in this study, all three original pharmacists moved on during the study but other staff remained constant. This highlights the importance of embracing the whole team in creating enduring change in a pharmacy environment. The whole pharmacy team approach has been recognised in related pharmacy practice research on suicide prevention (Gorton *et al.* 2019; Murphy *et al.* 2020).

Little change was perceived by clients, due to introduction of COVID-19 restrictions, the extended time between training (and subsequent changes) and follow-up. However, PWUD expressed positive comments about pharmacy staff – comparing them favourably to other pharmacies. This reflects previous research in which being treated with a positive attitude was valued (Laird *et al.* 2016).

This study found that training whole pharmacy teams in PIE was feasible and well received, and a further large trial is justified. The approach allowed staff to reflect on their behaviour and identify previous, potentially discriminatory practice. Attitudes compared favourably with previous national (Matheson *et al.* 2016) and local data (Laird *et al.* 2016) and demonstrated that the participating pharmacies were already well disposed to this patient group. Despite this, there were some aspects of their practice that were highlighted by training as being potentially stigmatising. The importance of clear and compassionate communication was evident. The increased mental health challenges for patients brought about by COVID-19 provided opportunities for staff to apply their new skills to this patient group and others. Development and extension of PIE training in community pharmacy is recommended with additional research on the potential knock-on impact for patients and client in terms of retention, harm reduction and recovery. Further research is required on the potential need for support and de-briefing for pharmacy staff who hear accounts of distressing events and life circumstances from patients and customers.

## Supplementary material

For supplementary material accompanying this paper visit http://doi.org/10.1017/S1463423623000087. click here to view supplementary material
