# Supplementary material for: Making community pharmacies psychologically informed environments (PIE): a feasibility study to improve engagement with people using drug services in Scotland
Source: Prim Health Care Res Dev. 2023 Mar 16;24:e20. doi: 10.1017/S1463423623000087 (PMC10050951; doi:10.1017/S1463423623000087)
Supplement: Supplementary file 1 [file S1463423623000087sup001.docx]

**Supplementary table: Attitude Responses**

| **Attitude statement** | **strongly agree**  **N**  **pre/post** | **Agree**  **N**  **Pre/post** | **Uncertain**  **N**  **Pre/post** | **Disagree**  **N**  **Pre/post** | **strongly disagree**  **N**  **Pre/post** |
| --- | --- | --- | --- | --- | --- |
| I believe dispensing Controlled Drugs to drug misusers, as part of a maintenance* programme, is part of a pharmacist’s professional remit. | 9/5 | 5/6 | 2/1 | 1/0 | 0/0 |
| I believe supplying or selling needles/syringes to intravenous drug misusers will help reduce the spread of HIV. | 12/8 | 1/3 | 2/0 | 2/0 | 0/0 |
| I believe drug misusers visiting my pharmacy would endanger the safety of staff. | 1/0 | 1/1 | 2/1 | 10/8 | 3/2 |
| I believe it is appropriate for pharmacists to provide advice (written or verbal) to drug misusers on the management of drug misuse. | 6/3 | 10/7 | 1/0 | 0/2 | 0/0 |
| I have no sympathy for drug misusers. | 0/0 | 0/0 | 0/0 | 7/3 | 10/9 |
| I would never supervise the consumption of Controlled Drugs by drug misusers on my pharmacy premises. | 0/0 | 0/0 | 0/0 | 3/3 | 14/9 |
| I believe providing drug misusers maintenance^^[[1]](#footnote-1)^*^ doses of Controlled Drugs won’t stop them using street drugs. | 5/2 | 5/1 | 4/9 | 3/0 | 0/0 |
| Drug misusers visiting my premises would have a damaging effect on business. | 0/1 | 0/1 | 4/0 | 8/8 | 5/2 |
| I believe needles and syringes should only be supplied to drug misusers through a syringe/needle exchange scheme. | 6/2 | 8/7 | 0/3 | 3/0 | 0/0 |
| I believe the community pharmacy is an appropriate place for a syringe/needle exchange scheme. | 6/3 | 7/7 | 3/1 | 1/1 | 0/0 |
| I believe supervising the consumption of Controlled Drugs by drug misusers on the pharmacy premises is an appropriate role for the community pharmacist. | 6/3 | 10/7 | 1/1 | 0/1 | 0/0 |
| I believe Controlled Drugs should be dispensed to drug misusers through a central clinic rather than community pharmacies. | 0/0 | 0/1 | 6/3 | 8/7 | 2/1 |
| I believe supervising the consumption of Controlled Drugs by drug misusers prevents the illicit selling of these Controlled Drugs on the street. | 0/2 | 2/1 | 5/4 | 6/5 | 3/0 |
| I would never provide advice (written or verbal) to drug misusers on the management of drug misuse. | 1/0 | 2/2 | 4/0 | 6/8 | 3/2 |
| I believe providing a syringe/needle exchange scheme is a good source of income for community pharmacies. | 3/3 | 8/4 | 3/5 | 2/0 | 0/0 |
| I believe drug misusers should only be prescribed Controlled Drugs if it is in reducing doses to help them ‘come off’ drugs. | 4/0 | 5/4 | 4/3 | 0/5 | 3/0 |
| I believe that if drug misusers ask to buy needles or syringes it indicates they are taking some responsibility for their health. | 4/1 | 5/3 | 4/5 | 3/3 | 0/0 |
| I believe the community pharmacy is not an appropriate place for a syringe/needle exchange. | 0/0 | 0/0 | 2/1 | 7/7 | 7/4 |
| *I believe it is unethical to sell drug misusers needles or syringes. | 6/1 | 0/3 | 5/3 | 2/4 | 4/1 |
| I believe dispensing Controlled Drugs to drug misusers is a good source of income for those pharmacies which dispense Controlled Drugs. | 2/3 | 7/3 | 5/6 | 2/0 | 0/0 |
| I believe providing drug misusers maintenance doses of Controlled Drugs will stop them using street drugs. | 0/1 | 0/1 | 3/2 | 8/6 | 5/2 |
| I believe providing maintenance doses of Controlled Drugs to drug misusers is a waste of NHS resources. | 0/0 | 1/0 | 3/2 | 7/7 | 5/3 |
| I would never provide advice (written or verbal) on safer injecting to intravenous drug misusers. | 0/0 | 1/2 | 1/1 | 7/4 | 7/5 |
| I believe Controlled Drugs should be dispensed to drug misusers through community pharmacies rather than a central clinic. | 0/1 | 6/3 | 6/5 | 4/3 | 0/0 |
| *I believe it is ethical to sell drug misusers needles or syringes. | 2/0 | 2/1 | 3/7 | 5/4 | 4/0 |
| I believe it is appropriate for pharmacists to provide advice to drug misusers on safer injecting. | 10/5 | 6/7 | 0/0 | 0/0 | 0/0 |

1. [↑](#footnote-ref-1)
